# Long Non-coding RNA: An Emerging Contributor and Potential Therapeutic Target in Renal Fibrosis

**DOI:** 10.3389/fgene.2021.682904

**Published:** 2021-07-27

**Authors:** Weiping Xia, Yao He, Yu Gan, Bo Zhang, Guoyu Dai, Feng Ru, Zexiang Jiang, Zhi Chen, Xiang Chen

**Affiliations:** Department of Urology, Xiangya Hospital, Central South University, Changsha, China

**Keywords:** long non-coding RNA, kidney disease, renal fibrosis, fibrosis, diabetic nephropathy

## Abstract

Renal fibrosis (RF) is a pathological process that culminates in terminal renal failure in chronic kidney disease (CKD). Fibrosis contributes to progressive and irreversible decline in renal function. However, the molecular mechanisms involved in RF are complex and remain poorly understood. Long non-coding RNAs (lncRNAs) are a major type of non-coding RNAs, which significantly affect various disease processes, cellular homeostasis, and development through multiple mechanisms. Recent investigations have implicated aberrantly expressed lncRNA in RF development and progression, suggesting that lncRNAs play a crucial role in determining the clinical manifestation of RF. In this review, we comprehensively evaluated the recently published articles on lncRNAs in RF, discussed the potential application of lncRNAs as diagnostic and/or prognostic biomarkers, proposed therapeutic targets for treating RF-associated diseases and subsequent CKD transition, and highlight future research directions in the context of the role of lncRNAs in the development and treatment of RF.

## Introduction

Renal fibrosis (RF) is a universal pathological process that leads to terminal renal failure in chronic kidney disease (CKD) (Sun et al., 2016); it is induced in response to diverse factors, such as external injury, inflammation, ischemia, hypoxia, myofibroblast activation and migration, and matrix deposition and remodeling (Liu et al., 2017; Humphreys, 2018; Bijkerk et al., 2019; Perry et al., 2019; Zhang S. et al., 2020). Many diseases are associated with RF development, including obstructive kidney disease, chronic glomerulonephritis, chronic pyelonephritis, systemic lupus erythematosus nephropathy, and hereditary nephropathies, such as Alport syndrome, diabetic nephropathy (DN), hypertensive nephropathy, and drug-induced nephropathy (González et al., 2008; Davidson, 2016; Seccia et al., 2017). However, the mechanism underlying RF development remains poorly understood, and existing treatments are ineffective. To understand the etiology and pathogenesis of RF and to delay or reverse disease progression, numerous researchers have investigated the mechanism underlying RF development. Wang Y. et al. (2019) demonstrated that inhibiting the expression of transient receptor potential melastatin-2 (TRPM2) might protect against RF and inflammation by preventing TGF-β1-mediated JNK activation. TGF-β can affect RF development *via* canonical or non-canonical TGF-β signaling pathways. Thus, anti-TGF-β treatment could ameliorate RF, but elucidating the exact mechanisms underlying RF development and identifying a treatment remain a challenge (Meng et al., 2016; Gu et al., 2020). Recently, long non-coding RNAs (lncRNAs) have attracted much attention. Accumulating evidence suggests that lncRNAs influence several biological processes, such as epithelial–mesenchymal transition (EMT), alternative splicing, proliferation, autophagy, apoptosis, and protein synthesis (Lu et al., 2017; Xu et al., 2017; Catana et al., 2020; Zhao X. et al., 2020). lncRNAs also play an important role in the progression and prognosis of many diseases (Yan et al., 2019; Shen et al., 2020; Wu et al., 2020). Wu et al. (2020) showed that knockdown of lncRNA SNHG17 in prostate cancer cell lines decreased proliferation, invasion, migration, and EMT transition capability while promoting apoptosis. Overexpression of lncRNA NEAT1 promotes proliferation, migration, and invasion and increases the EMT process in HeLa and SiHa cells (Shen et al., 2020). Similarly, several studies indicate that lncRNAs play a crucial role in the initiation and pathological progression of RF.

## Characteristics and Mechanisms of Action of LncRNA

Long non-coding RNA are defined as a class of endogenous non-coding transcripts with a length of over 200 nucleotides (Engreitz et al., 2016) that lack a specific integrated open reading frame and do not have protein-coding capacity (Kapranov et al., 2007; Yan et al., 2017). These transcripts usually have an mRNA-like structure; after splicing, lncRNAs have their own promoters, poly-adenosine tails, 5′ cap structures, and splice variants (Okazaki et al., 2002; Quinn and Chang, 2016). However, compared with those of protein-coding genes, the promoters of lncRNA are more conserved and the expression of these RNA occurs at relatively low levels (Derrien et al., 2012). In humans, lncRNAs regulate more than 70% of gene expression, with dynamic expression and varying molecular mechanisms underlying their ontogenesis, suggesting these RNAs play different biological roles and are involved in various disease processes. Unlike miRNAs that play a role at the post-transcriptional level, lncRNAs do not have a universal mode of action. Indeed, lncRNAs can bind RNA, DNA, or proteins as part of many biological processes (Dykes and Emanueli, 2017). Furthermore, lncRNA binding can be either enhanced or inhibited within an organism (Ignarski et al., 2019). Increasing studies on lncRNAs illustrate that these molecules play crucial roles in regulating specific cellular processes, such as modulating gene expression at the transcriptional, post-transcriptional, and epigenetic levels (Kung et al., 2013; Cao, 2014). Furthermore, the subcellular localization of lncRNA has an impact on their function and mechanism of action. For example, if an lncRNA is located in the cytoplasm, it can act as competing endogenous RNA (adsorbed miRNA), thereby affecting gene expression by regulating mRNA stability, degradation, and translation (Tay et al., 2014). Nuclear lncRNAs can regulate chromosome architecture, gene transcription, and rate of transcription to repress or activate gene expression (Yang Z. et al., 2019). Thus, lncRNAs are extensively involved in cell proliferation, survival, apoptosis, migration, and other cellular activities and play a vital role in biological process and disease progression.

**FIGURE 1:**
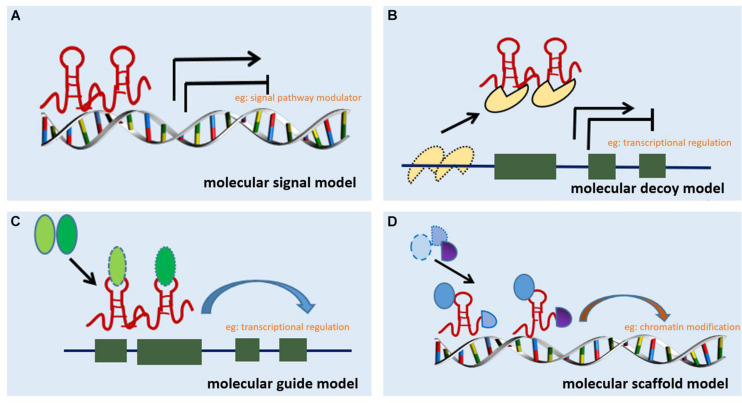
Mechanisms of action of lncRNA. **(A)** Molecular signal model: lncRNAs act as signaling pathway modulators to influence gene regulation in response to different stimuli. **(B)** Molecular decoy model: lncRNAs competitively bind to some RNA or proteins, causing the target to free a specific DNA region or protein, and then regulate the normal function. **(C)** Molecular guide model: lncRNAs interact with transcription factors or transcriptional regulators to guide transcription complexes to specific sites in the genome. **(D)** Molecular scaffold model: lncRNAs serve as a scaffold to bind two or more macromolecules (e.g., proteins, DNA, or other RNA species) and bring them into close proximity to chromatin.

Long non-coding RNA can be classified into six categories based on their location in the proximal protein-coding genes and the genome, i.e., bidirectional, intergenic, exon intronic antisense, natural antisense, sense overlapping, and intron sense overlapping (Ponting et al., 2009). Although studies on the mechanism of action of lncRNAs are limited, accumulating evidence indicates that lncRNAs regulate target gene expression through four main mechanisms. The first mechanism is represented by the molecular signal model (e.g., Figure 1A). lncRNAs exhibit cell-specific expression and distinct responses to different stimulating factors, indicating that the expression of lncRNAs is considerably controlled at the transcriptional level (Wang and Chang, 2011). Furthermore, lncRNAs respond to intra- and extracellular signaling pathways and act as modulators of signaling pathways. Previous studies have shown that lncRNAs are specifically transcribed under different stimuli *via* distinct signaling pathways and are involved in specific signal transduction processes (Mercer and Mattick, 2013). lncRNA can perform regulatory functions by functioning as signaling molecules without any involvement of protein translation. The second lncRNA mechanism is represented by the molecular decoy model (e.g., Figure 1B). After transcription, lncRNA can competitively bind to some RNA or proteins, thereby freeing a specific DNA region or target protein for other interactors (e.g., transcription factor or transcriptional regulator) and leading to the degradation of the target mRNA. Similarly, lncRNAs can bind to miRNAs, thereby eliminating the effects of miRNAs under physiological conditions, which would otherwise decrease or enhance mRNA stability to regulate the normal function of downstream target genes (Yao et al., 2019). For example, lncRNA MALAT1 can adjust pre-mRNA alternative splicing by trapping splicing factors inside nuclear speckles, whereas cytoplasmic lncRNAs can bind to miRNAs and derepress mRNA translation (Tripathi et al., 2010; Cesana et al., 2011). The third method represents the molecular guide model (e.g., Figure 1C). After transcription, lncRNAs interact with transcription factors and transcriptional regulators to guide transcription complexes to specific sites in the genome. Chromatin modifiers induce changes in local histone modifications to impact the expression of adjacent genes (Mercer and Mattick, 2013). Previous studies suggest that lncRNA-mediated transcriptional regulation can affect transcription, mRNA stability, or translation through homeopathic or *trans*-activation patterns (Wang and Chang, 2011). The last lncRNA mechanism represents the molecular scaffold model (e.g., Figure 1D). This is one of the most functionally intricate mechanisms of action of lncRNA, where lncRNA serves as a central platform to bind two or more macromolecules (e.g., proteins, DNA, or other RNA species) (Ignarski et al., 2019) and brings them into close proximity to chromatin. This mechanism achieves information convergence and integration between different signaling pathways, in a spatiotemporal manner, suggesting that lncRNAs play a central role in epigenetic processes as well as chromatin modification. Once we achieve a better understanding of the mechanisms underlying the abovementioned scaffolding role of lncRNA, whereby macromolecules are packaged in close proximity and expression is regulated, strategies can be designed to leverage specific signaling components to redirect and reshape cell function (Wang and Chang, 2011). A number of studies indicate that lncRNAs are also involved in the pathological process of RF, but a clear relationship among these processes remains unknown.

In this paper, we summarized recent studies on the role of lncRNAs in the pathogenesis of RF (Table 1). Understanding the precise mechanism of action of lncRNAs in RF may aid future studies and might be helpful for developing more effective therapies aimed at preventing RF.

**TABLE 1:** LncRNA dyregulated in RF diseases.

lncRNA categories	Main disease model/cell lines	Suggested function	Target gene/signaling	Function	Application	References
Erbb4-IR	UUO mouse kidney, TGF-β1 treated mTECs	Affect the transcription of collagen I and α-SMA	Smad7	Pro-RF	Therapeutic target for RF and T2DN	Feng et al., 2018
	T2DN mice (db/db), AGEs treated mTECs and mMCs	Transcriptional repression	miR-29b			Sun et al., 2018
HOTAIR	UUO rat’s kidney, TGF-β1 induced HK-2 cells and NRK-49F cells	miRNA binding	miR-124, Notch1/Jagged1 signaling pathway	Pro-RF	Therapeutic target for RIF	Zhou et al., 2019a,b
LINC00667	Human CRF tissues, CRF rat models, renal tubular epithelial cells	miRNA binding	miR-19b-3p	Pro-RF	Therapeutic target and prognostic marker in CRF	Chen et al., 2019
Arid2-IR	UUO mice kidney, TGF-β1 treated HK-2 cells	Affect interleukin-1β-NF-κB signaling and inflammatory cytokine	Smad3	Pro-RF	Therapeutic target for renal Inflammation	Zhou Q. et al., 2015
	HFD and STZ-induced diabetic mice/HG-induced mMCs	Affect the expression of ECM marks	–		Therapeutic target for DKD	Yang Z. et al., 2019
Blnc1	DN patient’s serum, STZ-induced DN rats, HG-induced HK2 cells	Affect inflammation, oxidative stress and RF	Nrf2/HO-1 and NF-κB pathways	Pro-RF	A potential therapeutic target in DN	Feng et al., 2019
NONHSAG053901	DN mouse model/MCs	Mediate renal inflammation	Egr-1/TGF-β pathway	Pro-RF	Diagnostic and therapeutic target in DN.	Peng and Huang, 2019
TCONS_00088786	UUO rat’s kidney, TGF-β1 induced NRK52E cells	miRNA binding	miR-132	Pro-RF	therapeutic target in RIF	Zhou et al., 2018
ARAP1-AS2	HG-induced HK2 cells	Participate in cytoskeleton rearrangement and EMT processes	–	Pro-RF	A pathogenic role in DN	Li L. et al., 2020
ATB	UUO model/TGF-β1 induced HK2 cells	Participate in EMT	–	Pro-RF	Therapeutic target in RF	Zhou and Jiang, 2019
Gm4419	db/db DN mice/HG treated MCs	Affect inflammation, fibrosis and proliferation	NF-κB/NLRP3 inflammasome signaling pathway	Pro-RF	Therapeutic target for DN	Yi et al., 2017
NR_033515	DN patient’s serum, HFD and STZ-induced diabetic kidney mice/HG-induced MMCs cells, BSA-stimulated HK2 cells	miRNA binding	miR-743b-5p	Pro-RF	A diagnostic and therapeutic target for DN	Gao et al., 2018
CHCHD4P4	Glyoxylate-treated mouse kidneys/COM treated HK-2 cells	Affect the EMT and cell proliferation	–	Pro-RF	A diagnostic and therapeutic target in calcium oxalate-induced kidney damage	Zhang et al., 2017
LOC105375913	Focal segmental glomerulosclerosis patient’s tubulointerstitial tissues/HK-2 cells	miRNA binding	miR-27b	Pro-RF	Therapeutic target for tubulointerstitial fibrosis	Han et al., 2019
MALAT1	STZ-induced DN/HG-induced mouse podocytes	Expression changed	Serine/arginine splicing factor 1 (SRSF1)	Pro-RF	A diagnostic and therapeutic target in RF diseases	Hu et al., 2017
	db/db DN mouse and HG-induced HK-2 cells	miRNA binding	miR-145-ZEB2 axis			Liu D. W. et al., 2019a
	UUO mouse, TGF-β1 induced HK2 cells	miRNA binding	miR-145-FAK axis			Liu et al., 2020
NEAT1	STZ-induced diabetic mice, HG-induced MMCs cells	Expression changed	Akt/mTOR signaling	Pro-RF	A pathogenic role in DN	Huang et al., 2019
	HFD- and STZ-induced DM mice, BSA treated HK2 cells	Expression changed	ERK1/2 signaling		Therapeutic target for DKD	Yang et al., 2020
	DN patient’s plasma, HG-induced MMCs cells	miRNA binding	miR-23c		Therapeutic target for DN	Li N. et al., 2020
PVT1	DN patient’s serum, HG-induced Mouse podocyte clone 5 (MPC5) podocytes and primary podocytes, STZ-induced DN mice	Expression changed	FOXA1	Pro-RF	Therapeutic target in DN	Liu D. W. et al., 2019
	db/db DN mouse, HG-treated MMCs	miRNA binding	miR-93-5p			Li J. et al., 2020
	DN patient’s serum, HG-treated MCs	miRNA binding	miR-23b-3p			Zhong et al., 2020
XIST	MN patient’s kidney biopsy, Ang II treated podocytes	miRNA binding	miR-217	Pro-RF	Diagnosis biomarker and therapeutic target for MM	Jin et al., 2019
	DN patient’s kidney, STZ-induced DN mice, HG treated HK-2 cells	miRNA binding	miR-93-5p		Therapeutic target for DN	Yang J. et al., 2019
1700020I14Rik	db/db DN mice/HG-treated MCs cells	miRNA binding	miR-34a-5p	Anti-RF	Therapeutic target in DN	Li et al., 2018
CYP4B1-PS1-001	db/db DN mice, HG-induced MMCs cells	Affect proliferation and fibrosis	–	Anti-RF	A biomarker for prognosis and a therapeutic target in DN	Wang et al., 2016a
	db/db DN mice, HG-induced MMCs cells	Protein binding	NCL			Wang S. et al., 2018
ENSMUST000 00147869	db/db DN mice/HG-induced MMCs cells	Affect proliferation and fibrosis	–	Anti-RF	Therapeutic target for DN	Wang et al., 2016b
MEG3	TGF-β1 induced HK2 cells	miRNA binding	miR-185	Anti-RF	Therapeutic target for RF	Xue et al., 2019
ZEB1-AS1	DN patient kidney biopsy, HG-induced HK2 cells	miRNA binding	miR-216a-5p	Anti-RF	Therapeutic target in DN	Meng and Zhai, 2020
	DN patient kidney biopsy, STZ-Induced DN mice, HG-induced HK2 cells	Expression changed	P53			Wang J. et al., 2018
ENST00000453774.1	Clinical RF specimens, UUO mice, TGF-β-induced HK-2 cells	Expression changed	Nrf2-keap1/HO-1/NQO-1 signaling	Anti-RF	Therapeutic target in RF	Xiao et al., 2019
NR03832	STZ-Induced DN Rats/HG treated HK-2 cells	miRNA binding	miR-324-3p	Anti-RF	Therapeutic target in DN	Ge Y. et al., 2019
MIAT	UUO, IRI kidneys	Affect myofibroblast formation	–	Pro-RF	Therapeutic target in RF	Bijkerk et al., 2019
	Human renal fibrotic tissues, UUO mice/TGF-β1 induced HK-2 cells	miRNA binding	miRNA-145	Pro-RF	Therapeutic target in RIF	Wang Z. et al., 2020
	STZ-induced diabetic rats/HG treated HK2 cells	Expression changed	Nrf2	Anti-RF	Therapeutic target	Zhou L. et al., 2015
GAS5	HG treated HK-2 cells	miRNA binding	miR-27a	Pro-RF	Diagnostic and therapeutic targets for DN	Lv et al., 2019
	TGF-β1 treated HK-2 cells and the kidneys of HDF/STZ mice	miRNA binding	miR-96-5p	Pro-RF	Therapeutic targeting in DKD	Wang W. et al., 2020
	HG-treated HK-2 cells	miRNA binding	miR-452-5p	Anti-RF	Therapeutic targeting in DN	Xie et al., 2019
	DN patients and HG treated MCs	miRNA binding	miR-221	Anti-RF	Therapeutic targeting for DN	Ge X. et al., 2019
	STZ treated DN rats	Recruiting EZH2 to MMP9 promoter region	MMP9 promoter region	Anti-RF	Therapeutic target in RF	Ge X. et al., 2019
TUG1	LPS treated HK2 cells	miRNA binding	miR-223	Anti-RF	Therapeutic target for lupus nephritis	Xu et al., 2018
	db/db DN mice, HG-treated NRK-52E cells	miRNA binding	miR-21		Therapeutic target for DN	Wang F. et al., 2019
	STZ-treated DN Rats, HG treated MMCs	Affect mesangial cells’ proliferation and ECM accumulation	PI3K/AKT pathway		Therapeutic target for DN	Zang et al., 2019
	HG treated human podocyte cell line (CIHP) cells	Affect podocytes apoptosis	ERS–CHOP–PGC-1α signaling	Pro-RF	Therapeutic target for DN	Shen et al., 2019
	Renal I/R rat, I/R injured HK2 cells	miRNA binding	miR-449b-5p		Diagnosis biomarker and therapeutic target for AKI	Xu et al., 2020

*Re = references; RF = renal fibrosis; Pro-RF = promoting renal fibrosis; anti-RF = anti-renal fibrosis.*

## Several LncRNAs Play a Promoting Role in RF

### LncRNA Erbb4-IR

Long non-coding RNA Erbb4-IR is a Smad3-related lncRNA that plays a role in kidney fibrosis (Zhou et al., 2014; Feng et al., 2018). This lncRNA is situated in the intron area of the *Erbb4* gene on chromosome 1 of mouse (Sun et al., 2018). In a unilateral ureteral occlusion (UUO) kidney model, Erbb4-IR was found to be highly upregulated in mouse tubular epithelial cells (mTECs). The level of lncRNA Erbb4-IR affected the transcription course of collagen I and α-SMA *via* a Smad3-related mechanism in TGF-β1-treated mTECs. Research has also revealed that *Smad7* is a target gene of Erbb4-IR and that specifically silencing the expression of Erbb4-IR resulted in the upregulation of renal Smad7, thereby blunting the TGF-β1/Smad3-induced RF *in vivo* and *in vitro* (Feng et al., 2018).

Advanced glycosylation end products (AGEs) induce RF through the TGF-β/Smad pathway by binding to either Smad7 or Smad3. Thus, these molecules can be potentially exploited as novel treatment modalities for DN (Chung et al., 2010). Chung et al. (2010) revealed that Erbb4-IR is highly upregulated after stimulation of mTECs and mouse mesangial cells (MMCs) with AGEs [rather than *via* high glucose (HG) *via* a Smad 3-dependent mechanism]. Furthermore, Smad3 knockdown blunted AGE-induced Erbb4-IR expression. Bioinformatic analysis revealed the presence of an Erbb4-IR binding site in the 3′ UTR of miR29b. Previous studies confirm that miR-29b protects against the progression of RF by regulating the TGF-β/Smad-dependent pathway under diabetic conditions. Additionally, miR-29b directly binds to the 3′ UTRs of collagen I and collagen IV, thereby suppressing RF (Xiao et al., 2012). Furthermore, Erbb4-IR knockdown in db/db mice resulted in reduced expression of collagen I and IV at mRNA and protein levels, an observation that may suggest effective future therapeutics for type 2 DN (T2DN) (Sun et al., 2018).

### LncRNA HOTAIR

Long non-coding RNA HOTAIR influences the progression of liver and myocardial fibrosis (Bian et al., 2017; Yu et al., 2017; Pan et al., 2018) while also being involved in the progression of renal interstitial fibrosis (RIF). Zhou et al. (2019a) showed that the overexpression of lncRNA HOTAIR downregulates miR-124 to activate the Notch1 pathway, thereby promoting EMT in TGF-β1-induced HK-2 cells and RIF in UUO rats. Paeonol reversed the effects of the HOTAIR/miR-124/Notch 1/Jagged1 axis on RIF and inhibited the effects of HOTAIR on EMT and migration of NRK-49F cells (Zhou et al., 2019b).

### LncRNA LINC00667

GSE37171 chip analysis revealed that lncRNA LINC00667 is upregulated in human chronic renal failure (CRF) tissues. The expression of TGF-β1, α-SMA, connective tissue growth factor (CTGF), and tissue inhibitor of metalloproteinase 1 at the mRNA and protein levels was also upregulated. A follow-up study showed that miR-19b-3p directly binds to lncRNA LINC00667 and CTGF. CTGF was upregulated and miR-19b-3p was downregulated in human CRF tissues. Additionally, miR-19b-3p overexpression offsets the positive profibrosis effect on lncRNA LINC00667 and the expression of fibrogenic factors in a CRF rat model (Chen et al., 2019). Thus, lncRNA LINC00667 is a potential therapeutic target and a novel prognostic marker in CRF.

### LncRNA Arid2-IR

Using high-throughput RNA-sequencing (RNA-Seq), 151 differently expressed Smad3-associated lncRNAs were found between the UUO kidney and Smad3 knockout mice (Zhou Q. et al., 2015). Smad3 plays an important role in renal inflammation and fibrosis, and a Smad3 binding site was located in a highly conserved region 1.6 kb upstream of Arid2-IR. Smad3 knockdown counteracted Arid2-IR upregulation in UUO kidneys. Thus, lncRNA Arid2-IR is a potential transcriptional target of Smad3. Further investigation clearly demonstrated that Arid2-IR knockdown in mTECs did not affect the TGFβ1-induced fibrotic process, including ECM marker expression, but inhibited NF-κB/p65 phosphorylation and interleukin-1β-mediated DNA binding, thereby inhibiting proinflammatory cytokine and chemotactic factor secretion. These results are consistent with the finding that *in vitro* deletion of Arid2-IR from UUO kidney cells has no effect on RF and the TGF-β/Smad3 pathway other than the inactivation of NF-κB signaling.

Transcription of lncRNA could be directly regulated by transcription factors. Yang Y. L. et al. (2019) confirmed that early growth response protein-1 (Egr1) is highly expressed in the kidneys of mice with diabetic kidney disease (DKD). Egr1, a transcription factor, enhances the proliferation rate and ECM production of mesangial cells (MCs) in DKD. Arid2-IR expression was significantly decreased after Egr1 knockdown, and Arid2-IR overexpression in HG-cultured MMCs was offset by Egr1 knockdown, thereby downregulating collagen 1 and α-SMA expression. Yang Y. L. et al. (2019) proved that Arid2-IR was also regulated by Egr1 in high-fat diet (HFD)-fed mice and in mice with streptozotocin (STZ)-induced diabetes. Nevertheless, more precise cross talks between Egr1 and Arid2-IR should be investigated.

### LncRNA Blnc1

Recent studies have demonstrated that the level of Blnc1 was increased in the serum of patients with DN and in rats with STZ-induced DN (Feng et al., 2019). Additionally, tissue damage and fibrosis were increased compared with those in the control group. Furthermore, Feng et al. (2019) showed that Blnc1 inhibition significantly reduced the level of fibrosis, inflammation, and oxidative factors *in vitro*. Additionally, HG injury in HK-2 cells significantly reduced the level of NRF2/HO-1 protein and activated the NF-κB pathway. These effects were reversed upon Blnc1 inhibition. Thus, Blnc1 serves as a novel regulator of inflammation, oxidative stress, and RF *via* the NRF2/HO-1 and NF-κB pathways in DN.

### LncRNA NONHSAG053901

Ho et al. (2016) confirmed that renal failure activates Egr-1, and Egr-1 deficiency alleviates TGF-β-induced renal inflammation and fibrosis. Concordantly, further investigations showed that lncRNA NONHSAG053901 binds directly to Egr-1. lncRNA NONHSAG053901 overexpression increased the expression of proinflammatory cytokines and RF biomarkers *via* the Egr-1/TGF-β pathway in MMCs. These effects were partially or fully restored upon co-transfection of siRNA against Egr1 (Peng and Huang, 2019). Thus, lncRNA NONHSAG053901 may serve as a promising target for antifibrotic therapies.

### LncRNA TCONS_00088786

To identify new potential molecular targets and biomarkers for RF, the strategy of high-throughput RNA-Seq followed by qRT-PCR was employed. This enabled the identification of differentially expressed RNA in urine and kidney tissue from rats after a 2-week UUO (Sun et al., 2017). The expression of 24 lncRNAs was upregulated and that of 79 lncRNAs was downregulated in the kidneys of UUO rats; the expression of 625 lncRNAs was upregulated and that of 177 lncRNAs was downregulated in the urine of UUO rats. In this study, TCONS_00088786—harboring a putative promoter containing a few conserved Smad3 binding motifs—was identified. Additionally, TCONS_00088786 was found to be dose- and time-dependently expressed in response to TGF-β induction and influence the expression of some fibrosis-related genes *via* a negative feedback loop in NRK52E cells.

Zhou et al. (2018) showed that the expression of lncRNA TCONS_00088786 was significantly increased in the UUO kidney *in vivo* and in TGF-β-treated NRK52E cells. Increased TCONS_00088786 levels upregulate the expression of miR-132, collagen I, and collagen III. Silencing of lncRNA TCONS_00088786 results in decreased expression of miR-132, collagen I, and collagen III. Therefore, this study demonstrated that lncRNA TCONS_00088786 contributes to the progression of interstitial fibrosis by upregulating the expression of miR-132. These findings indicate that TCONS_00088786 could definitely serve as a new therapeutic target for RIF.

### LncRNA ARAP1-AS2

Li L. et al. (2020) observed enhanced ARAP1-AS2 and ARAP1 expression in HG-stimulated HK-2 cells. Cdc42-GTP, cytoskeletal remodeling, cell viability, and migration were also increased in HG-treated HK-2 cells. Inhibition of ARAP1 expression counteracts the effects of HG and ameliorates RF. Furthermore, the overexpression of ARAP1-AS2 significantly increased EMT by positively regulating the expression of ARAP1. However, the mechanism by which ARAP1-AS2 regulates ARAP1 expression remains unclear. These results suggested that ARAP1-AS2/ARAP1 may affect RF *via* increased Cdc42-GTP levels in HG-treated HK-2 cells, a phenomenon that suggests new strategies to minimize DN progression (Li L. et al., 2020).

### LncRNA-ATB

Livin, a member of the anti-apoptotic protein family, is associated with the development, progression, and drug resistance of many human tumors *via* the stimulation of EMT (Li et al., 2013; Ge et al., 2016). Zhou and Jiang (2019) investigated the expression of livin in UUO models and TGF-β1-treated HK-2 cells and found that UUO elicits a high expression of livin and lncRNA-ATB. When livin was knocked-out using siRNA, the expression of lncRNA-ATB was significantly downregulated, thereby inhibiting TGF-β1-induced EMT in HK-2 cells. Hence, lncRNA-ATB and livin could serve as prominent therapeutic targets for RF.

### LncRNA Gm4419

Several studies demonstrate that the activation of NF-κB and NLRP3 is a critical link between inflammation and DN progression. Using RNA-Seq in the kidney tissues of db/db DN mice, Yi et al. (2017) identified 14 abnormally expressed lncRNAs, including lncRNA-Gm4419. Additionally, the expression of lncRNA Gm4419, p50, and NLRP3 inflammasomes was upregulated in MCs cultured in the presence of HG, and Gm4419 knockdown significantly downregulated the expression of proinflammatory cytokines and RF biomarkers in MCs. Furthermore, Gm4419 directly interacted with the p50 subunit of NF-κB to activate the NF-κB pathway. Additionally, the promoter region of the gene coding for the NLRP3 inflammasomes contains a p50 binding site in MCs. Overexpression of p50 or Gm4419 might increase the expression of NLRP3 inflammasomes, but Gm4419 overexpression did not alter the expression of proinflammatory cytokines and NLRP3 inflammasomes in MCs after transfection with SN50 (a p50-specific inhibitor) (Yi et al., 2017). Taken together, this study indicates that Gm4419 is a novel NF-κB-associated lncRNA, which activates the NF-κB/NLRP3 inflammasome signal *via* the interactions of p50 with Gm4419 and NLRP3 inflammasomes in MCs.

### LncRNA NR_033515

Gao et al. (2018) proposed that lncRNA NR_033515—whose expression is dramatically upregulated in the serum of patients with DN and is closely correlated with the different stages of DN—could serve as a crucial diagnostic and therapeutic target. lncRNA NR_033515 also plays a prominent role as a diagnostic marker of DN. *In vitro* studies demonstrated that increased NR_033515 expression in MMCs promotes the expression of PCNA and cyclin D1; upregulates ASK1, FN, α-SMA, and P38 expression; and is positively associated with the expression of EMT biomarkers (E-cadherin and vimentin) *via* miR-743b-5p (Gao et al., 2018). Further investigations are needed to validate the potential interactions between NR_033515 and miR-743b-5p in DN.

### LncRNA CHCHD4P4

When renal tubules become injured due to calcium oxalate crystal deposition, EMT occurs in the epithelial cells of renal tubules, thereby initiating RF. In total, 376 lncRNAs were differentially expressed between the glyoxylate-exposed and healthy mice kidney groups (Zhang et al., 2017). Further analysis of the human and mouse lncRNAs, i.e., CHCHD4P4 homologs—which were identified in mice and humans using BLAST—revealed a 425-bp-long lncRNA located on chromosome 3 (Zhang et al., 2017). Calcium oxalate monohydrate (COM) induced the overexpression of CHCHD4P4 in HK-2 cells. Silencing of CHCHD4P4 resulted in the inhibition of mesenchymal-like morphological features and decreased the transcription of vimentin, zinc finger E-box binding homeobox1 (ZEB1), and Snail. More importantly, CHCHD4P4 overexpression inhibited cell proliferation by promoting the apoptosis of HK-2 cells treated with COM, suggesting that CHCHD4P4 might aid the early diagnosis and treatment of kidney disease. Additional research is necessary to explore the mechanism by which CHCHD4P4 regulates the expression of EMT-related genes.

### LncRNA LOC105375913

In the tubulointerstitial tissue of patients with focal segmental glomerulosclerosis (FSGS), the level of LOC105375913 was significantly increased and positively correlated with the tubulointerstitial fibrosis score (Han et al., 2019). Overexpression of LOC105375913 in HK-2 cells increased the expression of FN, collagen I, and Snail at mRNA and protein levels in HK-2 cells and in the tubular cells of patients with FSGS. Bioinformatic analysis and RNA pull-down revealed that LOC105375913 functions as ceRNA and competitively binds to miR-27b, thus regulating Snail expression and causing tubulointerstitial fibrosis in mice and in C3a-stimulated HK-2 cells. p38 and the transcription factor XBP-1s regulate LOC105375913 expression in HK-2 cells. Overexpression of XBP-1s or p38 also increases the level of endogenous LOC105375913, promotes the binding of miR-27b to LOC105375913, and increases the expression of fibrosis markers in HK-2 cells. Conversely, this binding between LOC105375913 and miR-27b was significantly inhibited upon XBP-1s knockdown or p38 inhibition, and this resulted in decreased expression of fibrosis markers in HK-2 cells (Han et al., 2019).

### LncRNA MALAT1

MALAT1 is also known as mascRNA because it is located in the cell nucleus with a cytoplasmic tRNA-like small RNA (Zong et al., 2016). Hu et al. (2017) showed that the expression of lncRNA MALAT1 is dramatically increased in the background of STZ-induced DN when proteinuria was present and is correlated with by HG-induced podocyte damage. MALAT1 knockdown enhances the integrity of podocyte architecture and function by downregulating SRSF1 expression and reduces the nuclear accumulation of β-catenin triggered by HG. Furthermore, β-catenin also regulates MALAT1 expression due to its ability to bind to the MALAT1 promoter region. Downregulated expression of the β-catenin gene decreases MALAT1 expression, while MALAT1 regulates the pattern of post-transcriptional β-catenin splicing. These results demonstrate the feedback loop mechanism that exists between β-catenin and MALAT1 during podocyte damage (Hu et al., 2017). Furthermore, the expression of MALAT1 is increased in the plasma and kidney tissues of patients with acute kidney injury, in hypoxic kidney biopsies of mice, and in cultured and *ex vivo*-sorted hypoxic endothelial and HK-2 cells (Kölling et al., 2018). Liu B. et al. (2019) provided more evidence for the molecular mechanisms underlying EMT and RF in DN. In their study, MALAT1 was significantly upregulated, while miR-145 was downregulated in the renal tissues of DN mice. They proposed that MALAT1 functions as a “sponge” for miR-145 and subsequently upregulates the expression of its target gene *ZEB2* to promote EMT and fibrosis in HK-2 cells cultured in HG medium. Furthermore, Liu et al. (2020) found that m6A is the primary methyltransferase, which induces lncRNA MALAT1-exacerbated renal fibrogenesis in obstructive nephropathy *via* the miR-145/FAK signaling pathway. Taken together, lncRNA MALAT1 may be a potential biomarker for the diagnosis and treatment of RF in CKD.

### LncRNA NEAT1

Long non-coding RNA NEAT1 is a pivotal regulator of the mitogen-activated protein kinase (MAPK) pathway in human lupus disease (Zhang et al., 2016). In STZ-induced diabetes mellitus (DM) rats and glucose-treated MMCs, NEAT1 is significantly upregulated. Interestingly, the proliferation of MMCs and fibrosis in DN are inhibited by NEAT1 siRNA *via* activation of Akt/mTOR signaling (Huang et al., 2019). As an anti-aging protein, Klotho markedly alleviates renal tubular EMT and inhibits NEAT1 expression during DN development. Silencing NEAT1 in BSA-induced HK-2 cells can reverse the protective effect caused by Klotho *via* ERK1/2 signaling (Yang et al., 2020). These results are consistent with those obtained for STZ- and HFD-treated DN mice. To further investigate the pivotal role of NEAT1 in DN, Li N. et al. (2020) demonstrated that lncRNA NEAT1 expression was dramatically elevated in the serum of patients with DN. As described above, these findings suggest a new regulatory pathway involving NEAT1, which might be a potential therapeutic target for DKD.

### LncRNA PVT1

Plasmacytoma variant translocation 1 (PVT1) is a famous lncRNA regulator in DM (Hanson et al., 2007; Alwohhaib et al., 2014). PVT1, a 1.9-kb-long lncRNA, mediates the overexpression of ECM proteins in DN (Alvarez and DiStefano, 2011) and was the first lncRNA reported to be related to kidney disease (Alvarez et al., 2016). Accordingly, PVT1 is upregulated in the serum of patients with DN (Liu D. W. et al., 2019; Zhong et al., 2020). Similarly, PVT1 expression significantly increased in mouse podocyte clone-5 and in primary podocytes in an HG environment (Liu D. W. et al., 2019). Moreover, PVT1 recruits the enhancer of Zeste homolog 2 (EZH2) to facilitate the recruitment of H3K27me3 to the FOXA1 promoter area, thus downregulating FOXA1 expression to promote apoptosis and podocyte damage in DN (Liu D. W. et al., 2019).

PVT1 is also overexpressed in the serum of patients with DN, kidneys of mice with DN, and HG-induced MMCs or human MCs (hMCs). PVT1 knockdown inhibits cellular migration, invasion, proliferation, and fibrosis. Furthermore, PVT1 knockdown blocks the PI3K/Akt/mTOR signal and promotes the apoptosis of MMCs under HG conditions by upregulating miR-93-5p (Li J. et al., 2020). Furthermore, Zhong et al. (2020) validated that silencing PVT1 might relieve HG-induced FN and α-SMA expression and proliferation in hMCs by inhibiting the NF-κB pathway *via* the miR-23b-3p/WT1 axis.

### LncRNA XIST

Aberrant expression of lncRNA XIST is closely related to the inactivation of the X chromosome. This lncRNA is a well-known tumor suppressor gene or oncogene in many tumors, but little research has been conducted regarding its role in RF. Huang et al. (2014) were the first to report significantly increased lncRNA XIST levels in the urine of patients with membranous nephropathy (MN); this increase in lncRNA XIST levels in the urine is positively related to disease severity. They also found that XIST is regulated by H3K27me3 levels in the kidney of mice with MN. These results provide new insights into the diagnosis and treatment of MN. Furthermore, lncRNA XIST functions as a ceRNA for miR-217 to facilitate Toll-like receptor 4 (TLR4) expression, thus inducing the apoptosis of podocytes (Jin et al., 2019). Furthermore, lncRNA XIST levels are increased in the kidneys of patients with DN, in HG-treated HK-2 cells, and in mice with DN. XIST knockdown inhibits RIF in DN by repressing cyclin-dependent kinase inhibitor 1A and increasing miR-93-5p expression (Yang J. et al., 2019). Further studies based on the results of the abovementioned studies are warranted before lncRNA XIST can be used as a novel clinic biomarker for patients with DN.

## Several LncRNAs Play an Antifibrosis Role in RF

### LncRNA 1700020I14Rik

Long non-coding RNA 1700020I14Rik (ENSMUST00000147425) is located in chromosome 2 (Chr2: 119594296–119600744). Li et al. (2018) demonstrated that this lncRNA 1700020I14Rik functions as a miRNA sponge and competitively binds to miR-34a-5p. While other lncRNAs were significantly downregulated in HG-cultured MCs and in db/db DN mice, lncRNA 1700020I14Rik displayed the highest sequence conservation in the cells and mice compared with their homologous sequence in humans. Furthermore, overexpression of lncRNA 1700020I14Rik reduces the proliferation and expression of fibrosis markers (Col-4, FN, TGFβ1) of MCs in an HG environment by directly interacting with miR-34a-5p, which inhibits the Sirt1/HIF-1α signaling pathway. These changes were reversed by miR-34a-5p mimics. Thus, lncRNA 1700020I14Rik probably serves as an important therapeutic target for DN.

### LncRNA CYP4B1-PS1-001

Long non-coding RNA CYP4B1-PS1-001 (transcript ID: ENSMUST00000118753) is located on the reverse strand of chromosome 10 and is significantly downregulated in db/db mouse models (Wang et al., 2016a). Moreover, dose-dependently decreased CYP4B1-PS1-001 expression was further confirmed in MMCs under different glucose levels. Overexpression of CYP4B1-PS1-001 altered the expression of proliferation indexes and fibrosis markers in MMCs during the progression of DN (Wang et al., 2016a). Further studies demonstrated that CYP4B1-PS1-001 promoted nucleolin (NCL) ubiquitination and degradation, thereby inhibiting the fibrosis process of MMCs and indicating that the CYP4B1-PS1-001/NCL axis might be a prognostic biomarker and effective therapeutic target for the treatment of DN (Wang S. et al., 2018).

### LncRNA ENSMUST00000147869

ENSMUST00000147869, which is 629 nt long and located on chromosome 4, is downregulated in the renal tissues of DN mice (Wang et al., 2016b). Overexpression of ENSMUST00000147869 reduces the expression of fibrosis markers and proliferation indexes in MMCs under HG conditions. Wang et al. (2016b) provided new insights into the pathogenesis and development of DN.

### LncRNA MEG3

Long non-coding RNA MEG3 is situated on the human chromosome 14q32 (Wylie et al., 2000). Xue et al. (2019) found that MEG3 is significantly downregulated in TGF-β1-treated HK-2 cells. MEG3 overexpression significantly decreases the expression of mesenchymal markers and increases the expression of epithelial markers. Moreover, miR-185 regulates the expression of DNA methyltransferase 1 (DNMT1), which thereby regulates the expression of MEG3 *via* modulation of CpG methylation in the MEG3 promoter in TGF-β1-induced HK-2 cells (Xue et al., 2019). Thus, the miR-185/DNMT1/MEG3 pathway is a new mode of regulation for RF and further verification of its role in RF is needed.

### LncRNA ZEB1-AS1

Long non-coding RNA ZEB1-AS1, which is transcribed from a shared bidirectional promoter with ZEB1 (Gao et al., 2019), is a cancer-related and antifibrotic lncRNA. When expressed, this gene acts as an oncogenic regulator in many human tumors (Ni et al., 2020). Meng and Zhai (2020) demonstrated that ZEB1-AS1 overexpression inhibits HG-induced RF by suppressing EMT and fibrogenesis. Mechanistically, they identified that ZEB1-AS1 impedes RF by regulating BMP7 expression and inhibits EMT of HK-2 cells by competitively binding to miR-216a-5p (Meng and Zhai, 2020). Several studies reported that BMP7, which plays a key role in many renal diseases, is a direct target of miR-216a-5p. Importantly, as predicted by bioinformatics analysis and confirmed by the luciferase reporter assay, the expression levels of fibrosis-related proteins and EMT-related markers reduced significantly with the overexpression of ZEB1-AS1 in the HG-treated HK-2 cells. The expression of these markers is restored by targeting miR-216a-5p and downregulating BMP7 in HK-2 cells (Meng and Zhai, 2020).

Previous findings showed that p53 is significantly upregulated in STZ-induced mice with DN (Peng et al., 2015). Inhibition of p53 expression may ameliorate TGF-β-induced RF induced in UUO (Overstreet et al., 2014; Peng et al., 2015). Wang J. et al. (2018) found that p53 is mainly expressed in the renal tubular cells of db/db mice at 16 weeks of age. Furthermore, they reported that p53 inhibitors and the deletion of p53 in the proximal tubule ameliorate interstitial fibrosis in db/db mice and in STZ-induced DN mice. Furthermore, p53 physically interacts with the promoter region of lncRNA ZEB1-AS1. They also verified that lncRNA ZEB1-AS1 binds to the H3K4 methyltransferase MLL1 and promotes H3K4me3 histone modification in the ZEB1 promoter, which negatively regulates ZEB1 expression in HK-2 cells and causes ECM accumulation (Wang J. et al., 2018). Taken together, these findings provide evidence that p53-lncRNA ZEB1-AS1 and the ZEB1 axis may be new potential therapeutic targets for RF in DN.

### LncRNA ENST00000453774.1

Long non-coding RNA microarray profiling was used to detect lncRNA dysregulation in TGF-β-treated HK-2 cells. The results show that lncRNA ENST00000453774.1 (lncRNA 74.1) is dramatically downregulated, consistent with the results obtained from clinical RF specimens (Xiao et al., 2019). Autophagy and oxidative stress are closely related in RF. Interestingly, overexpression of lncRNA 74.1 promotes ROS defense mechanisms *via* the Nrf2-keap1/HO-1/NQO-1 pathway by accelerating pro-survival autophagy and decreasing the expression of ECM markers, collagen I, and FN. These effects were found to alleviate RF and therefore represent a potential treatment for renal diseases (Xiao et al., 2019).

### LncRNA NR_038323

LncRNA_NR038323 is located on chromosome 8 (Chr8: 23336208–23366125). Ge Y. et al. (2019) demonstrated that lncRNA NR_038323—which is localized in the cytoplasm—plays an antifibrotic role in HG-treated HK-2 cells and is induced in response to a 24–72-h HG treatment. However, HG-triggered increase in endogenous lncRNA NR_038323 expression was unable to limit HG-induced expression of collagen I, collagen IV, and FN. Interestingly, this effect was almost completely reversed upon overexpression of lncRNA NR_038323 in HG-treated HK-2 cells. lncRNA NR_038323 contains the binding sites for miR-324-3p and DUSP1, suggesting that this lncRNA is a direct target of miR-324-3p. Increased expression of lncRNA NR_038323 suppressed miR-324-3p expression, which resulted in increased expression of DUSP1, a phenomenon that resulted in inhibition of the ERK1/2 and p38MAPK signal in HG-induced RF (Ge Y. et al., 2019). Furthermore, *in vivo* studies showed that lncRNA NR_038323 overexpression mediates antifibrotic effects by regulating the miR-324-3p/DUSP1 pathway in patients with DN and in rats with STZ-induced DN (Ge Y. et al., 2019). These findings suggest lncRNA_NR03832 can serve as a potential therapeutic target for DN.

## LncRNAs With a Double-Edged Sword Role in RF

### MIAT

MIAT, also known as Gomafu, was first detected in mitotic progenitors and post-mitotic retinal precursor cells (Rapicavoli et al., 2010). This lncRNA decreases myofibroblast formation and alleviates the progression of kidney fibrosis. Bijkerk et al. (2019) observed marked expansion of pericyte-derived myofibroblasts in the interstitium of UUO 10 days after UUO. However, 2 days after unilateral ischemia–reperfusion injury (IRI), no clear expansion was observed. MIAT, which critically influences myofibroblast formation, is highly increased in myofibroblasts isolated from IRI and UUO kidneys (Bijkerk et al., 2019). Moreover, knockdown of MIAT suppresses myofibroblast formation, as evidenced by the decreased expression of α-SMA, collagen 1, Smad2, and Smad3. MIAT is also upregulated in UUO mice and human clinical kidney specimens. In TGF-β1-induced HK-2 cells, MIAT knockdown counteracts the effect of TGF-β1 on the EMT process in cells by interacting with the miRNA-145/EIF5A2 axis (Wang Z. et al., 2020).

However, in STZ-induced diabetic rats, MIAT expression is decreased and negatively correlated with the expression of serum creatinine and blood urea nitrogen (Zhou L. et al., 2015). Besides, *in vitro* studies show that the increased expression of MIAT caused by pcDNA-MIAT plasmid transfection reverses the inhibitory action of Nrf2 expression and improves renal tubule cell viability (Campo and Mylotte, 1988; Zhou L. et al., 2015). Hence, elucidating the role of these cellular pathways in the pathophysiology and modulation in different types of RF could help reverse the pathological process of RF.

### LncRNA GAS5

GAS5 expression is increased in HK-2 cells in an HG environment. Moreover, Lv et al. (2019) revealed that silencing GAS5 alleviates the HG-mediated reduction in HK-2 cell viability and apoptosis by downregulating miR-27a and BNIP3 and inactivating the JNK pathway. In STZ/HDF mouse kidneys and TGF-β1-treated HK-2 cells, GAS5 is highly expressed. GAS5 knockdown relieves renal tubular epithelial fibrosis by regulating the antifibrotic miR-96-5p, which inhibits FN1 expression (Wang W. et al., 2020).

In contrast, Xie et al. (2019) demonstrated that GAS5 expression is decreased in HK-2 cells treated with HG and that GAS5 overexpression suppresses oxidative stress, inflammation marker expression, and pyroptosis by directly targeting miR-452-5p in HG-induced HK-2 cells. Furthermore, GAS5 is downregulated in patients with DN and MCs (Ge X. et al., 2019). Overexpression of GAS5 inhibits proliferation, causes G0/1 phase arrest, and alleviates the expression of FN, collagen 4, and TGF-β1 in MCs. In addition, GAS5 upregulates SIRT1 expression and inhibits cell proliferation and fibrosis by acting as a ceRNA for miR-221. In another study, GAS5 was shown to recruit EZH2 to the matrix metalloproteinase 9 (MMP9) promoter regions, downregulating MMP9 and alleviating RIF and inflammatory reactions in STZ-induced DN rats (Zhang L. et al., 2020).

### LncRNA TUG1

Long non-coding RNA TUG1, which is situated at chromosome 22q12, was first described as an important component of retinal development and photoreceptor function in mouse retinal cells. TUG1 was significantly decreased in HK-2 cells after lipopolysaccharide treatment. TUG1 overexpression was shown to protect renal tubular epithelial cells from inflammatory injury by downregulating miR-223 and upregulating Sirt1 expression, resulting in PI3K/AKT activation and NF-κB inactivation. The study further describes the protective anti-inflammatory effects of TUG1 in lupus nephritis (Xu et al., 2018). TUG1 overexpression promotes the expression of TIMP3 *via* the regulation of miR-21, ultimately inhibiting HG-stimulated NRK-52E cell fibrosis and RF in DN mice. These findings provide evidence for a new approach for DN fibrosis treatment (Wang F. et al., 2019). Furthermore, TUG1 is downregulated in DM rats and in HG-induced MCs. Finally, overexpression of TUG1 suppresses the proliferation and ECM deposition of MCs, which is caused by high-level glucose induction *via* PI3K/AKT pathway inhibition (Zang et al., 2019).

Podocytes have been observed to be very prone to damage in diabetes. Shen et al. (2019) observed that TUG1 is upregulated in HG-treated human podocyte cells. Moreover, TUG1 improves endoplasmic reticulum stress, thereby influencing podocyte apoptosis by modulating the C/EBP homologous protein (CHOP) and peroxisome proliferator-activated receptor-gamma co-activator 1 alpha (PGC-1α) signaling pathways in HG-induced developing DNs. Accumulating evidence shows that overexpression of high mobility group box 1 (HMGB1) aggravates renal ischemia/reperfusion injury by promoting inflammatory responses in mice (Chen et al., 2017). TUG1 is also upregulated in renal ischemia/reperfusion (I/R) injury models. Silencing TUG1 reduces I/R-induced inflammation and apoptosis by directly regulating miR-449b-5p, HMGB1, and matrix metalloproteinase 2 expression (Xu et al., 2020).

## The Function of LncRNAs in RF

**FIGURE 2:**
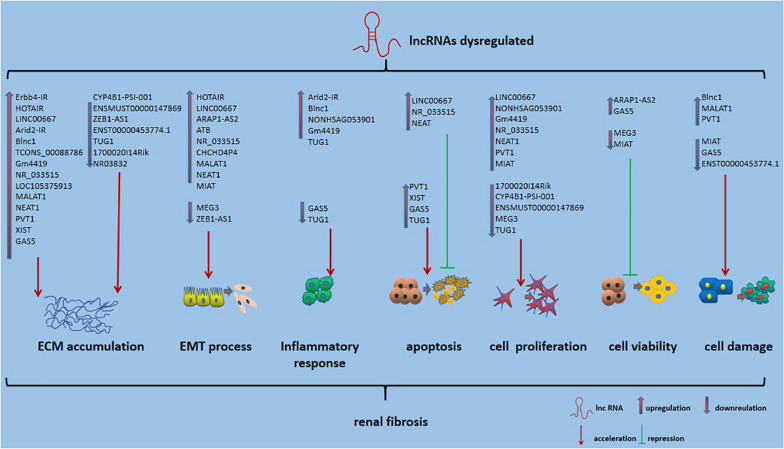
Role of lncRNAs in RF. Also shown is that lncRNA could affect ECM accumulation, EMT process, inflammatory responses, apoptosis, cell proliferation and cell damage, and cell viability in RF.

Long non-coding RNAs, which influence various biological processes in RF, have been emerging as key regulators. Here, we briefly summarize some currently known regulatory mechanisms by which lncRNAs are involved in RF, especially in DN (Figure 2). ECM accumulation, the EMT process, inflammatory responses, apoptosis, cell proliferation and cell damage, and cell viability have been shown to be involved in mechanisms underlying RF development.

## Conclusions and Prospects

Numerous studies indicate that lncRNAs, whose expression is aberrant in pathways involved in kidney disease, are rapidly emerging as potential therapeutic targets and diagnostic markers. However, their roles in kidney fibrosis are scarcely understood, and further studies are required to elucidate the molecular mechanisms underlying their functions. For example, at least three lncRNAs, Blnc1, ENST00000453774.1, and MIAT, affected the expression of Nrf2 (Zhou L. et al., 2015; Feng et al., 2019; Xiao et al., 2019). In the DN and UUO models, lncRNA expression was negatively correlated with Nrf2 expression. Moreover, Arid2-IR and NONHSAG053901, which could directly bind to Egr-1 and positively regulate Egr-1 expression, were increased in the DN model (Peng and Huang, 2019; Yang Y. L. et al., 2019). Different lncRNAs might play similar pro/anti-RF roles by binding to the same transcription factor or protein in distinct RF diseases or in the same RF disease. However, the mechanisms of interaction between different lncRNAs and regulation of aberrant cross talks in RF require elucidation. Other confounding factors in our understanding of lncRNAs include the fact that lncRNAs can have more than one mechanism of action in RF. For example, GAS5 can not only influence EZH2 recruitment to the MMP9 promoter region and competitive binding of miR-96-5p in the DN model (Wang W. et al., 2020; Zhang L. et al., 2020) but also activate or repress their target genes. This phenomenon is extremely common, which suggests that novel mechanisms are yet to be elucidated.

Furthermore, lncRNA/miRNA interactions are a common regulatory strategy in RF, but the gene regulatory network is complex and is yet to be fully elucidated. For example, PVT1 and XIST could bind to miR-93-5p at the predicted site; silencing of PVT1 or XIST inhibits the fibrosis process in HG-treated MMCs or HK-2 (Yang J. et al., 2019; Li J. et al., 2020). Similarly, MALAT1 and MIAT could influence cell proliferation, viability, migration, and the EMT process by binding to miR-145 in TGF-β1-stimulated HK-2 cells (Liu B. et al., 2019; Liu et al., 2020; Wang Z. et al., 2020). Clinical trials on lncRNA/miRNA are still underway. Researchers believe that the therapeutic targeting of lncRNA/miRNA might elicit fewer off-target effects due to the precision of the combination (Zhao Z. et al., 2020). However, whether the combination of two or more different lncRNAs targeting the same miRNA or protein-coding gene, or the combination of two or more different miRNA targeting the same lncRNA, will increase the risk of targeted therapy off-targets is presently unclear. Moreover, with respect to RF, no relevant research report on whether simultaneous intervention of these differentially expressed lncRNAs would have a synergistic or counteracting effect on the same downstream target gene exists. Hence, more comprehensive studies on lncRNA involving CRISPR/Cas9 DNA and CRISPR/Cas13 RNA editing techniques and alternative splicing are required to provide insights into the specific mechanisms by which dysregulated lncRNA function in RF. Furthermore, lncRNA inhibition or overexpression *in vivo* without any accompanying toxic side effects, particularly in human renal tissue, remains challenging. Therefore, urologists should cooperate with molecular biologists working in the fields of materials science, to enable detailed functional verification in multiple models of RF and to identify lncRNA with clinical application potential. Only by overcoming these issues can we identify additional RF-related lncRNAs. Finally, lncRNAs are expected to serve as promising targets for antifibrotic therapies.

## Author Contributions

WX, XC, and ZC developed the original content and drafted the manuscript. WX, XC, ZC, YH, YG, BZ, and GD contributed to article acquisition. FR and ZJ contributed to the preparation of the table. All authors contributed to the article and approved the submitted version.

## Conflict of Interest

The authors declare that the research was conducted in the absence of any commercial or financial relationships that could be construed as a potential conflict of interest. The reviewer ZX declared a shared affiliation, with no collaboration, with the authors to the handling editor at the time of review.

## Publisher’s Note

All claims expressed in this article are solely those of the authors and do not necessarily represent those of their affiliated organizations, or those of the publisher, the editors and the reviewers. Any product that may be evaluated in this article, or claim that may be made by its manufacturer, is not guaranteed or endorsed by the publisher.
